# Additional vertebrae are imaged in 44% of pediatric patients when limited CTs are ordered: a pilot study

**DOI:** 10.1007/s43390-026-01464-1

**Published:** 2026-06-08

**Authors:** Robert G. Watkins, Vivien Chan, Christopher Watterson, Elaine Butterworth, Kenneth D. Illingworth, David L. Skaggs

**Affiliations:** 1 Department of Orthopaedic Surgery, Cedars Sinai Medical Center, 444 S San Vicente Blvd Suite 901, Los Angeles, CA 90048 USA; 2 Department of Diagnostic Radiology Cedars Sinai Medical Center, Los Angeles, CA USA; 3 Alberta Children’s Hospital, University of Calgary, Calgary, Alberta Canada

**Keywords:** Computed tomography, Pediatric spine, Radiation exposure, Imaging protocols

## Abstract

**Purpose:**

To determine how often physician-ordered limited CTs in pediatric spine patients are not followed, and if this leads to excess radiation.

**Methods:**

Single-center retrospective review of a tertiary pediatric spine practice. Inclusion: CT scans ordered for specific vertebrae (e.g., L4-5) Exclusion: generalized regions (cervical, thoracic, lumbar). Demographics, CT orders and scans, and radiation data were obtained from electronic records. A vertebra was considered imaged only if the entire vertebral body was imaged. Analyses used descriptive statistics, chi-square tests, and one-way ANOVA with Tukey-adjusted post-hoc pairwise comparison.

**Results:**

93 patients (mean age 16.1 ± 3.4) with 121 CT scans met criteria. In 53 (44%) CTs, more vertebrae were imaged than ordered, yielding a mean of 2.1 times more radiation. Scans occurred at 30 sites. A tertiary medical center with a new limited CT protocol performed 61 (50.4%) scans, with 49.2% scanning additional vertebrae. A private network of 3 outpatient imaging centers with established imaging protocols performed 26 scans, with 3.8% scanning additional vertebrae. The remaining 34 scans were performed at 23 external sites, with 64.7% scanning additional. The imaging center was significantly associated with additional vertebrae imaging frequency (X^2^ = 23.6, *P* = 0.0002).

**Conclusion:**

44% of patients with limited CT scans of the spine had additional vertebrae scanned compared to what was ordered. This corresponded to an estimated mean of twice the intended radiation dose. Limited CTs at centers with established imaging protocols do not image additional vertebrae in > 96% of patients. Pediatric spine centers should look to develop specific limited CT protocols to help decrease radiation exposure.

## Introduction

Computed Tomography (CT) is an imaging technique that provides visualization of tissues and is commonly used for spinal pathology. CT has become one of the most commonly ordered studies in medicine due to its rapidity, availability and effectiveness, however, CTs substantially increase patient radiation exposure, accounting for nearly 60% of the radiation exposure in medical imaging. This is especially concerning in children due to their developing organs, small body size, and large amount of overlying tissue [1–3]. To address these concerns, pediatric radiology has emphasized the as low as reasonably achievable (ALARA) principle and the need to keep radiation doses as low as possible, especially in children. In practice, this includes tailored imaging protocols that minimize *z*-axis coverage and avoid over scanning. In spine imaging specifically, limited CT scans isolated to the area of pathology are used. Unfortunately, deviations from ordered vertebral coverage, whether due to technologist discretion or limitations in the original order, may result in unnecessary radiation exposure. This effect is compounded for patients who require multiple CT scans [4–8]. Complex spinal pathologies may require special imaging protocols, such as three-dimensional reconstruction and thin-cut CTs (< 1 mm) for preoperative planning and use of spinal navigation. When these protocols are not performed as ordered, patients may require repeat CT scans to obtain the necessary images, further exposing these patients to excessive radiation.

Although previous work has described radiation risks of pediatric CT and technical dose-reduction strategies [8, 9], there is limited data on how often spine-specific limited CT orders are not followed in routine practice, how much additional radiation is delivered when orders are not followed, and how these patterns differ across imaging environments. This gap is particularly relevant for pediatric spine surgeons and radiologists who care for patients requiring repeated CT imaging. This study therefore aims to quantify the frequency and types of order deviations for limited spine CTs, estimate the additional radiation associated with these deviations, and explore differences in adherence to limited CT orders between centers. We hypothesize that pediatric limited spine CTs frequently exceed the ordered vertebral region, thereby exposing patients to additional radiation.

## Methods

This is a single-center retrospective study of a tertiary pediatric spine practice. The study period was April 2021 to April 2025. Patients were included if they had a confirmed or suspected spinal pathology (e.g., spondylolysis, fractures, or spondylolisthesis) and underwent a CT scan with an order specifying a limited number of vertebral bodies to be imaged (e.g., L4-L5). Patients were excluded if the CT scan was ordered for a generalized region (cervical, thoracic, lumbar). Patients were excluded from analysis of slice thickness if the scan was taken on a SPECT-CT scanner. All CT scans meeting the inclusion criteria during this interval were identified through an electronic search of electronic medical records (EMR) and manual review of radiology reports by a full-time research fellow who was trained by an attending neurosurgeon. This yielded 93 patients with 121 eligible CT scans. Because this was an exploratory, hypothesis-generating study focused on characterizing practice patterns, a priori sample size calculation was not performed; instead, we analyzed all available eligible scans during the study period. Demographic characteristics were summarized using standard descriptive statistics.

The following data were obtained from patient electronic EMR: age, sex, CT date, CT location, slice thickness ordered, vertebral levels ordered, and whether a 3D reconstruction was ordered. Patient CT scans were reviewed to abstract vertebral levels, slice thickness, and 3D reconstruction status. A scan was considered concordant with the order if the imaged vertebral levels exactly matched the levels specified in the order. Any mismatched levels (more, fewer, or different vertebrae) was classified as a vertebral-level error, regardless of clinical justification. Deviations in slice thickness or 3D reconstruction relative to the order were recorded as separate error types; scans could have more than one error. Regarding vertebral-level errors, a vertebra was only counted as imaged if the entirety of the vertebral body was in view. Scans were evaluated for compliance with the order regarding vertebral levels, slice thickness, and 3D reconstruction. Additionally, scanner-reported CTDIvol (mGy) and dose length product (mGy·cm) (DLP) were abstracted from the dose report for each scan. Estimated expected DLP was calculated by multiplying the number of vertebral levels specified in the order by the per-level DLP (actual DLP divided by the number of vertebrae imaged). Estimated excess DLP was defined as the difference between the actual DLP and the expected DLP. We did not adjust DLP for pitch of convert to organ or effective dose; instead, DLP values are used as relative indexes to compare radiation exposure across scans and imaging centers. Indications were abstracted from the imaging order and grouped into categories based on primary diagnostic intent. Orders lacking a clear indication were classified as “not specified.” Imaging centers were categorized as (1) tertiary medical center (2) a private network of outpatient imaging centers with an imaging protocol for limited CT scans, and (3) external imaging centers.

Descriptive statistics were used for analysis. Chi-square tests were conducted to evaluate the association between the frequency of additional vertebrae scanned and (1) imaging center and (2) implementation of the ordering panel. One-way ANOVA tests were conducted to examine whether the imaging center was associated with (1) the percentage of additional radiation exposure received by patients who had extra vertebrae scanned and (2) the CTDI used. Alpha level was 0.05 for significance. For chi-square analyses, expected cell counts we inspected, and it was confirmed that all expected counts exceeded 5, supporting the validity of the chi-square approximation. For ANOVA models, assumptions were evaluated by visual inspection of residual and Q-Q plots and by Shapiro–Wilk tests for normality and Levene tests for homogeneity of variances. Although Shapiro–Wilk (*p* = 0.015) and Levene (*p* = 0.007) tests indicated some deviation from ideal assumptions, Welch’s ANOVA and Kruskal–Wallis tests yielded similar conclusions, so the one-way ANOVA and Tukey-adjusted pairwise comparisons are presented as the primary analyses. Some patients underwent more than one CT scan during the study period; analyses were performed at the scan level, treating each scan as an independent observation. We acknowledge this clustering as a limitation and address it in the Discussion.

We conducted an IRB-approved retrospective chart review with a waiver of consent and HIPAA authorization, as the study posed no more than minimal risk and involved no patient interaction, with identifiable data accessible only to approved study personnel and destroyed when no longer needed. Therefore, patients were not informed about use of their data.

## Results

The distribution of indications is shown in Table 1. The most common indication was back or neck pain, followed by postoperative assessment. A substantial proportion of studies lacked a clearly documented indication.

**Table 1:** Distribution of indications

Indication	Count (*n*, %)
Pain (non-specific)	23 (19.0%)
Postoperative evaluation	20 (16.5%)
Pars defect/spondylolysis spectrum	17 (14.0%)
Scoliosis/deformity	9 (7.4%)
Fracture/trauma	8 (6.6%)
Spondylolisthesis/anterolisthesis	4 (3.3%)
Preoperative planning	4 (3.3%)
Transitional/congenital variant	3 (2.5%)
Not specified	33 (27.3%)

### Vertebrae, slice thicknesses, and 3D reconstruction

93 patients (mean age 16.1 ± 3.4 years) with 121 CT scans over a 4-year period met the inclusion criteria for this study. Of the 121 scans, 53 (44%) scanned more vertebrae than ordered. For these scans, a mean of 3 ± 2.5 additional vertebrae were scanned with a range of 1 to 10 vertebrae. Among scans with additional vertebrae imaged, 52.8% included 1–2 extra vertebrae, 26.4% included 3–5, and 20.8% included ≥ 6 extra vertebrae. Two scans imaged less vertebrae than ordered and two scans imaged the incorrect vertebrae, totaling to 57 (47.1%) scans that did not follow the order according to the vertebrae specified. 65 scans (53.7%) did not follow the CT order in at least one respect (levels, thickness, 3D reconstruction). Only 55 (45.5%) orders required thin-cut (1 mm or less) images, 4 (7.3%) of which did not perform thin-cut images. Of the 121 scans, 95 ordered 3D reconstruction. Of these 95 orders, 15 (15.8%) of these scans did not perform 3D reconstruction (Table 2). Of the 65 scans with any deviation from the order, 18 (27.7%) had more than one type of error (e.g., both additional vertebrae and lack of ordered 3D reconstruction).

**Table 2:** Deviations from order

Error	Frequency (*n*, %)
Scan more levels than ordered	53/121 (44%)
Scan fewer levels than ordered	2/121 (1.7%)
Scan correct number of levels, wrong section	2/121 (1.7%)
Incorrect slice thickness	4/55 (7.3%)
No 3D reconstruction when ordered	15/95 (15.8%)

### Radiation doses

The mean DLP of all scans was 276 mGy-cm. The mean CTDIvol was 14.5 ± 7.5 mGy. The mean estimated expected DLP was 207 mGy-cm. Only scans with more vertebral levels imaged than ordered resulted in excess estimated DLP. For the 53 (44%) scans that met this criterion, the mean estimated excess DLP was 163 mGy·cm. Patients in these scans received an estimated 2.1 times the expected radiation from the order.

### Comparison between facilities

**Fig. 1:**
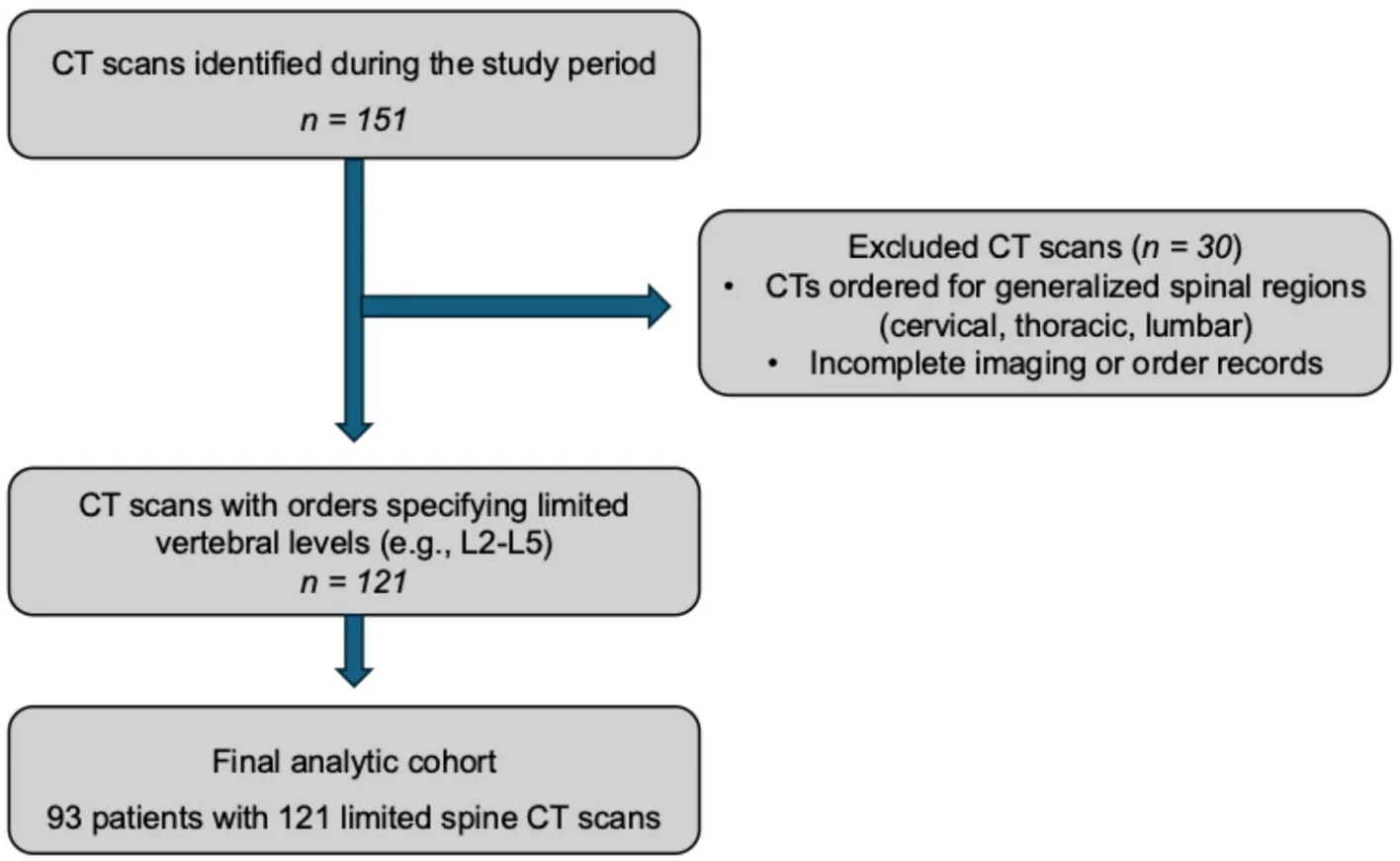
STROBE-style flow diagram of cohort selection

The 121 scans were performed at 30 different locations. The tertiary medical center performed the most scans, 61 (50.4%), followed by external sites with 34 scans (28.1%), and a private network of outpatient imaging centers with an imaging protocol for limited CT scans with 26 scans (21.5%). External sites had the highest frequency of additional vertebrae imaged 64.7%, followed by the tertiary medical center at 49.2%. The private network of outpatient imaging centers had the lowest error rate at 3.8% (Table 3). A chi-square test of independence indicated a significant association between imaging center and error frequency, X^2^ = 23.6, *p* < 0.001. For patients who were exposed to excess radiation (i.e., had additional vertebrae scanned), external locations had a mean of 2.45 times the intended radiation. The tertiary medical center had a mean of 1.89 times the intended radiation, and the private network of outpatient imaging centers had a mean of 1.33 times the intended radiation. For patients who had additional vertebrae scanned, a one-way ANOVA test indicated a significant difference between imaging center and the percent of additional radiation patients were exposed to, *F*(2,54) = 6.81, *P* = 0.0023. A one-way ANOVA test indicated a significant difference between imaging center and the CTDI used, *F*(2, 118) = 5.16, *p* = 0.007. Post-hoc Tukey-adjusted pairwise comparisons showed that external imaging centers delivered significantly higher ratios of actual DLP to intended DLP compared to the tertiary medical center (difference = 0.56, *p* = 0.041). No other pairwise differences between imaging centers were statistically significant Fig. 1.

**Table 3:** Scan accuracy by location

Location	*n* (%) *n* = 121	Frequency of additional vertebrae imaged (n, %)	% additional radiation	Mean CTDI (mGy)
Tertiary medical center	61 (50.4%)	30/61 (49.2%)	188.8%	14.07 ± 6.3
External	34 (28.1%)	22/34 (64.7%)	245.2%	12.25 ± 7.7
Private, local imaging center	26 (21.5%)	1/26 (3.8%)	133.3%	18.24 ± 7.4

## Discussion

In this cohort of pediatric spine patients, nearly half of physician-ordered limited CTs imaged more vertebrae than requested, resulting in an estimated mean radiation dose of about twice what would have been expected if the orders had been followed. This estimate reflects imaging order deviation rather than a measured increase in clinical risk. These findings confirm our hypothesis that limited spine CTs frequently exceed the ordered vertebral region and show that even modest deviations from the prescribed z-axis can substantially increase radiation exposure.

Children are significantly more sensitive to ionizing radiation than adults due to rapid cell division, ongoing organ development, and longer lifespans [10]. Because of this, the ALARA principle has been adopted, especially in high-dose scans such as CTs [11]. Even slight increases in radiation exposure carry long-term risks, so it is crucial to follow ALARA and avoid unnecessary scanning. The best way to do this is to adhere to the ordered protocol, avoiding excess vertebrae scanned and repeat scans from incorrect slice thickness or lack of 3D reconstruction. Imaging precision is crucial in diagnosis and preparation for surgery as 3D reconstruction provides important spatial information and fine slice thickness (≤ 1 mm) increases resolution and is required for use of navigation using preoperative imaging. In this cohort, deviations most commonly involved scanning beyond the requested vertebral levels, with fewer instances of incomplete or incorrect-level coverage, underscoring that careful adherence to the ordered z-axis is a key and immediately actionable target for total dose reduction.

For the 53 scans in which excess vertebrae were scanned, the mean estimated excess DLP was 163 mGy-cm. Published pediatric CT conversion factors indicate that an excess DLP of approximately 160 mGy-cm corresponds to an additional effective dose on the order of 1–3 mSv, depending on patient size and spinal region [12]. Although such estimates are approximate, they are commonly used to contextualize relative radiation burden and highlight the potential impact of modest z-axis extensions in children undergoing imaging [8]. The increased radiation burden is particularly concerning given the cumulative exposure many pediatric patients receive from follow up imaging over time [3]. In the context of scoliosis, where serial imaging is often required, even small deviations from the order may compound and elevate lifetime cancer risk [3, 10, 11]. For spondylolysis, low-dose CT protocols have been developed, resulting in decreased radiation exposure [11]. Fadell et al. found that limiting the z-axis coverage and only scanning the relevant anatomy decreased radiation exposure and increased consistency among reviewers as scans were less ambiguous [13]. Brady et al. found that CT deep learning reconstruction (DLR) both improved image quality and reduced radiation dose [14]. As CT technology and practices continue to develop—becoming more efficient in providing a clear image while exposing the patient to less radiation—it is essential both ordering providers and technologists work diligently to use only the imaging parameters necessary for diagnostic accuracy.

It is important to recognize that not all deviations from the written order represent inappropriate imaging. Technologists may deliberately extend coverage by a level to avoid missing pathology, compensate for uncertain localization, or include adjacent structures, improving diagnostic quality and potentially avoiding the need for repeat imaging [15, 16]. In addition, some orders may be nonspecific or incomplete, making it difficult for technologists to determine the exact range that the ordering provider intended. In this study, any order that met these criteria was excluded. Any mismatch between ordered and imaged vertebral levels was classified as an error for the purposes of quantifying order deviations and estimating associated dose, without adjudicating the clinical appropriateness of each deviation. Our findings should therefore be interpreted as highlighting opportunities for joint protocol refinement and clearer communication between surgeons, radiologists, and technologists, rather than as a pure measure of technologist performance.

The performed chi-square, ANOVA, and Tukey-adjusted post-hoc pairwise comparison tests suggest that scan accuracy and the amount of excess radiation a patient is exposed to due to over scanning are associated with imaging location, as external sites demonstrate a higher rate of additional vertebrae and excess radiation error than the medical center or private network. The higher rate of error associated with external locations may reflect the lack of specialization in pediatric imaging, inconsistent access to spine specific imaging protocols, or a larger disconnect between imaging center and provider. Private imaging centers may benefit from closer collaboration with referring providers and more detailed or consistent imaging protocols. The imaging protocol for limited spine CT scans of the private network of imaging centers is added as Appendix 1. This protocol places emphasis on the CT technologist verifying that the CT scan will be limited, verbally confirming this to the patient, and then only scanning 0.5 vertebra above and below the requested region. These precautions may be what led to the consistency and low error rate seen in the private network of imaging centers, thus other institutions may consider implementing similar protocols.

However, this measure does not account for potential excess radiation dose due to the usage of a high CTDIvol. CTDIvol is a measure of the average dose per slice, accounting for information such as pitch, which is protocol specific [12]. Although the private network scarcely scanned more vertebrae than ordered, they also implemented the highest mean CTDI, as seen in our results. As CTDI directly affects DLP, and thus effective radiation dose, the use of a high CTDI further exposes patients to radiation that may be deemed unnecessary, especially if the lower CTDI values used by other imaging centers are adequate. Therefore, ordering physicians, radiologists, and technologists should look to determine specific protocols that limit both scanning beyond the intended range and the use of high CTDIvol values, in accordance with the ALARA principle.

This study is limited by its analysis at the scan level, which did not account for clustering of multiple scans within individual patients. If one patient had multiple CT scans at different times, they were analyzed as independent scans. This approach may have underestimated variance and influenced *p* values, thus, the findings in the differences between imaging centers should be interpreted with caution. Radiation exposure was estimated using scanner-reported dose metrics rather than direct measurement of organ or effective dose. In addition, data were derived from a single tertiary pediatric spine practice, which may limit generalizability to other practice settings.

Standardizing imaging protocols across imaging locations, enhancing communication between ordering physicians and technologists, and implementing clear ordering panels may reduce the frequency of over scanning and the use of CTDIvol values and their associated radiation burden. Future studies across multiple centers with larger pediatric cohorts could validate these findings and comparisons between the effects of increased z-axis range versus increased CTDIvol values could provide insight into the creation of standard imaging protocols.

## Data Availability

Data available from the corresponding author on reasonable request.

## Appendix

Private network limited CT protocol.

## Private network of imaging centers limited CT spine imaging protocol

### Purpose

To ensure consistency, appropriateness, and radiation dose optimization when performing limited CT examinations of the spine at specifically requested vertebral levels.

### Scope

This protocol applies to all limited CT spine examinations in which the referring provider specifies one or more vertebral levels (e.g., L5 only).

### Protocol description

1. Order verification and patient communication
2. When a limited CT study is ordered for specific vertebral level(s), the CT technologist shall verify the requested level(s) as documented in the imaging order.
3. Prior to image acquisition, the technologist will inform the patient and/or family that a *limited study* is being performed and will verbally confirm the vertebral level(s) requested, using standardized script language (e.g., “This is a limited CT study of L5.”).
4. Scan coverage parameters
5. For a single-level request, the standard acquisition range shall include **0.5 vertebral levels above and 0.5 vertebral levels below** the requested level to ensure adequate anatomic context while minimizing unnecessary radiation exposure.
6. For example, when **L5** is specified, the scan range shall extend from the **mid-body of L4 through the mid-body of S1**.
7. Technologist discretion and escalation
8. If there is uncertainty regarding the requested level(s), scan extent, or clinical indication, the technologist shall consult the supervising radiologist prior to image acquisition.
9. Routine expansion of scan coverage beyond this protocol is discouraged unless explicitly authorized by a radiologist.

### Rationale

Limited CT examinations require active protocol oversight and collaboration between radiologists and technologists. In the absence of defined protocols, default or “autopilot” scanning practices may result in imaging beyond the clinical need. This protocol ensures precision, consistency, and adherence to ALARA principles.
